# Fixation stability and deviation in optical coherence tomography angiography using soft contact lens correction in myopes

**DOI:** 10.1038/s41598-021-91403-z

**Published:** 2021-06-03

**Authors:** Andrew Kwok-cheung Lam, Kenny Kin-hei Lau, Ho-yin Wong, Jasmine Pui-kwan Lam, Man-for Yeung

**Affiliations:** 1 Center for Myopia Research, School of Optometry, The Hong Kong Polytechnic University, Kowloon, Hong Kong SAR China; 2 Center for Eye and Vision Research (CEVR), Kowloon, Hong Kong China

**Keywords:** Health care, Medical imaging

## Abstract

To compare fixation deviation and stability with soft contact lens correction and device built-in auto-focus system during optical coherence tomography angiography (OCTA). This observational study measured OCTA metrics first with contact lens correction, followed by removal of contact lenses, using the device auto-focus system at a University Optometry Clinic, Hong Kong. All participants were habitual soft contact lens wearers with either low or high myopia. OCTA measurements were obtained using a spectral domain OCTA. Fixation deviation was distance (in pixels) of the fovea to the center of the OCTA measurement grid. Fixation stability was test–retest repeatability (TRR) and coefficient of variation (CV) of fixation deviation from three consecutive OCTA measurements. OCTA metrics included vessel length density (VD), perfusion density (PD), and foveal avascular zone (FAZ) area. Averaged OCTA metrics were calculated from three measurements and compared between the two correction methods. The mean ± SD spherical equivalent of 74 eyes from 74 myopes measured was − 1.94D ± 0.75D in low myopes (n = 37) and − 7.97D ± 1.31D in high myopes (n = 37). When corrected with contact lenses, visual acuities of high myopes (median [IQR], − 0.06 [0.08] logMAR) and low myopes (− 0.02 ± 0.08 logMAR) were similar (P = 0.060), and with similar fixation deviation (5.0 ± 2.2 pixels vs 5.3 [3.6] pixels; P = 0.689). High myopes had poorer fixation stability than low myopes (TRR: 10.2 pixels vs 7.5 pixels; CV: 65% vs 54%, respectively). The worst fixation stability occurred when high myopes were corrected using the auto-focus system (TRR: 12.5 pixels, CV: 72%). The difference in VD and PD was within 1 mm^−1^ and 1%, respectively. The FAZ area was similar. Difference in OCTA metrics was small in each refractive group (< 1 mm^−1^ in VD, and < 2% in PD). High myopes had more stable fixation when corrected when wearing contact lenses. Subjects with good contact lens corrected visual acuity should wear their contact lenses during OCTA measurements.

## Introduction

Due to its non-invasive nature, optical coherence tomography (OCT) has become a standard imaging modality in ophthalmic practice. Optical coherence tomography angiography (OCTA), an advancement of OCT technology, can compare multiple OCT images at the same location. Any temporal motion of scattering particles in tissue from sequential OCT images represents blood flow. Vasculature of the retina, choroid, and optic disc region can be derived by detecting blood flow of en face OCT images in different layers^1^. Although OCTA can provide non-invasive evaluation of the retinal vasculature, currently it cannot replace conventional imaging modalities such as fluorescein angiography^2,3^ and indocyanine green angiography^4^.

One major limitation of OCTA is that the image quality is affected by common artefacts, including projection, segmentation errors, defocus, and motion^5–7^. Some artefacts can be controlled by examiners, such as better image focus, while others rely on improved algorithms of proprietary software. Balasubramanian et al.^8^ reported that a two-diopter defocus during OCT could significantly reduce peripapillary retinal nerve fiber layer thickness by 10 μm. An accurately focused image is also very important in OCTA. One diopter of defocus was found to reduce vessel area density, or perfusion density (PD) by 6%^9^. An additional diopter of defocus could further reduce PD by 2%. Such reduction was due to reduced signal strength from defocus. Yu et al.^10^ found that PD also decreased linearly with signal strength across different OCTA platforms.

Most OCT and OCTA devices have built-in auto-focus system to compensate for patients’ refractive errors to acquire clear images. Patients are usually unaided during OCT and OCTA acquisitions, with their refractive errors corrected using the auto-focus system. Berkenstock et al.^11^ found that soft contact lens correction could improve signal strength in OCT scans. Peripapillary retinal nerve fiber layer thickness was found to be increased through contact lens correction. Their study included 20 eyes from only 12 patients. Aviram et al.^12^ did not find any difference in OCT image quality nor macular thickness results with and without contact lens wear. However, subjects had only low to moderate amount of myopia. Most built-in auto-focus systems are limited to correct spherical defocus. Jung et al.^13^ found that vessel length density (VD) could be reduced by 0.5 mm^−1^ per diopter of induced with-the-rule astigmatism.

If refractive error is not fully corrected, not only will blurry OCT images be acquired, but poor location of the fovea may also occur. Pak et al.^14^ reported that a decentration greater than 200 μm from the fovea could lead to OCT foveal thickness at the central 1-mm zone being reduces by 9 μm. Kim et al.^15^ moved the measurement location artificially to simulate gaze instability. A horizontal shift greater than 59 μm or a vertical shift greater than 47 μm could reduce the ganglion cell-inner plexiform layer thickness significantly.

OCTA measurement at the macula region is usually presented according to the Early Treatment of Diabetic Retinal Study (ETDRS) format. When fixation is not at the foveal center, measurement results at different ETDRS subfields may not be accurate. When fixation location varies at different acquisitions, it is hard to conclude if the difference in OCTA metrics is due to an ocular health issue or simply measurement errors from fixation instability. We hypothesized that high myopes could have more stable fixation through soft contact lens correction compared with that obtained using the built-in auto-focus system.

## Methods

Healthy adult myopes with habitual soft contact lens wear were recruited. This included low and high myopia according to the spherical equivalent (sphere plus half the refractive astigmatism). Low myopes had a spherical equivalent ≥ − 3D and high myopes had a spherical equivalent ≤ − 6D. Subjects with a history of ocular diseases, contact lens complications, or eccentric fixation were excluded.

This study adhered to the tenets of the Declaration of Helsinki and was approved by the Institute Research Board of The Hong Kong Polytechnic University (HSEARS20190318002). Informed consent was obtained from each subject before eye examination commenced. All subjects visited the campus optometry clinic with their contact lenses in situ. Ophthalmic examinations included habitual contact lens corrected visual acuity (CL-VA) using a logMAR chart, over-refraction using an auto-refractor (Nidek ARK-510A, Nidek Co. Ltd., Japan). OCTA measurement was then conducted (protocol described below). Thereafter, subjects were required to remove their contact lenses. After measuring their unaided vision, non-cycloplegic auto-refraction and axial biometry (Nidek AL-Scan, Nidek Co. Ltd., Japan), OCTA measurements were conducted again using the same OCTA device and following the same protocol. Refractive errors of the subjects were corrected using the built-in auto-focus system. The eye with the better CL-VA was chosen from each subject for OCTA measurements, or randomly selected if CL-VA was the same for both eyes.

### Image acquisition

OCTA measurements were obtained using a commercially available spectral-domain OCT (Cirrus 5000, Carl Zeiss Meditec, Inc., CA, USA). This spectral-domain OCT uses a superluminescent diode laser at 840 nm wavelength, with a scan speed of 68 k A-scans/second. The Cirrus AngioPlex software uses an optical microangiography complex algorithm (OMAG) to identify changes in the phase and intensity of the OCT scans to quantify motion contrast (10.0 software version). Valid OCTA images had to achieve signal strength of at least 7^16^, and no obvious artefacts. The superficial capillary plexus was determined using the AngioPlex software to locate the internal limiting membrane at the outer boundary of the inner plexiform layer. A 6 × 6 mm pattern with 350 × 350 pixels was chosen. This provides a 17.1 μm transverse resolution. Triplicate OCTA scans centered at the fovea were obtained in each eye using the two correction methods, i.e. contact lens and auto-focus. FastTrac retinal tracking function was turned on for each OCTA measurement to reduce motion artefacts. However, the “track to prior scan” option was not used so each scan was independent to the others^17^. Artificial tears (0.4% sodium hyaluronate, Aquadrop + , Precilens) were applied, if necessary, to prevent ocular surface dryness.

### Statistical analysis

**Figure 1:**
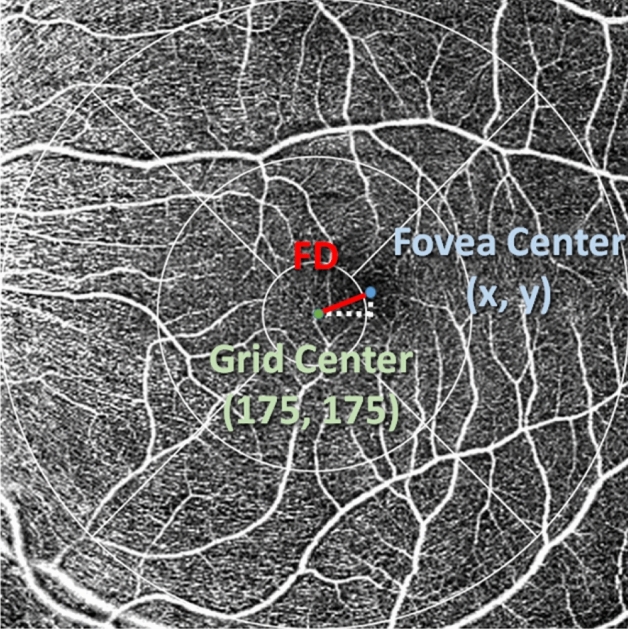
Coordinates of foveal center are x, y. Fixation deviation (FD) in pixel was calculated as distance between grid center and foveal center using the formula below. FD = $\sqrt{{{\left(175-x\right)}^{2}+\left(175-y\right)}^{2}}$

Each OCTA measurement was exported and the XML file included location (x–y coordinates) of the fovea. From a 350 × 350 pixels scan, the center of the scan has a coordinate of 175,175. Fixation deviation (FD) was defined as deviation of the fovea (in pixels) from the center of the 350 × 350 scan area. Figure 1 shows the calculation of fixation deviation. An average FD from three consecutive OCTA measurements was calculated for each eye and the average FD of all subjects was computed. Fixation stability refers to test–retest repeatability (TRR) of FD from the three consecutive OCTA measurements. TRR of all subjects was calculated from the within-subject standard deviation times 2.77^18^. Coefficient of variation (CV) of fixation deviation, the ratio of the standard deviation to the mean, from three OCTA measurements was also calculated. TRR and CV were used to indicate fixation stability of the two refractive groups.

It has been recommended that averaging three OCTA metrics is better than taking a single OCTA measurement^19^. In each eye, OCTA metrics (VD, PD, and foveal avascular zone (FAZ) area), using the Early Treatment of Diabetic Retinal Study (ETDRS) format, were averaged from three OCTA measurements. Results were compared between the two correction methods.

All statistical analyses were performed using SPSS version 26.0 (IBM Corp. USA). Measurement results were tested for normality using the Shapiro–Wilk test. Parametric tests (paired and unpaired t-tests) and non-parametric tests (Wilcoxon and Mann–Whitney tests) were used accordingly. Data are presented as mean ± standard deviation (SD) or median (interquartile range, IQR).

## Results

Seventy-four eyes of 74 myopes (age 18 to 47 years) were included. Thirty-seven subjects had low myopia. Table 1 shows the demographic data of the subjects. The two myopic groups had similar age. Although high myopes had greater refractive astigmatism, they had similar CL-VA. Twenty-five high myopes (68%) were wearing toric soft contact lenses, versus 15 (41%) of low myopes.

**Table 1:** Demographic data (Mean ± SD, or median (interquartile range)).

	Low myopes	High myopes	Analysis
Age (years)	20.6 ± 1.6	21 (3)	Mann–Whitney test, p = 0.162
Gender	20 male & 17 female	20 male & 17 female	Chi square, p = 0.592
Auto-refraction in SEQ (D)	− 1.94 ± 0.75	− 7.97 ± 1.31	Unpaired t-test, p < 0.001
Refractive astigmatism (D)	− 0.78 ± 0.46	− 1.88 ± 1.00	Unpaired t-test, p < 0.001
Axial length (mm)	24.45 ± 0.0.87	26.85 ± 0.98	Unpaired t-test, p < 0.001
Contact lens corrected visual acuity (logMAR)	− 0.06 (0.08) Range: 0.22 to − 0.14	− 0.02 (0.08) Range: 0.20 to − 0.22	Mann–Whitney test, p = 0.06
Over-refraction in SEQ (D)	− 0.125 ± 0.367	− 0.284 ± 0.571	Unpaired t-test: t = 1.433, p = 0.159

*SEQ* spherical equivalent, sphere + ½ astigmatism, *logMAR* Logarithm of the Minimum Angle of Resolution.

Table 2 shows the fixation deviation and stability results of the two myopic groups. Low myopes had similar fixation deviation regardless of correction methods (contact lens: 5.0 ± 2.2 pixels; auto-focus: 4.4 ± 2.1 pixels). Compared with low myopes, high myopes had greater variation in fixation deviation (contact lens: IQR 3.6 pixels; auto-focus: IQR 6.1 pixels). Fixation deviation of the two groups was similar when corrected with contact lenses (Mann–Whitney test, p = 0.689). Signal strength was higher in low myopes compared with high myopes (Mann–Whitney test, p = 0.041). When corrected using the auto-focus system, high myopes had greater fixation deviation than low myopes (Mann–Whitney test, p = 0.029). Signal strength was also significantly higher in low myopes compared with high myopes (Mann–Whitney test, p < 0.001). Fixation stability, in terms of TRR and CV, was poor in high myopes especially when using the auto-focus system. CV of different pairs did not show significant difference (two-sample coefficient of variation tests, all p > 0.05).

**Table 2:**
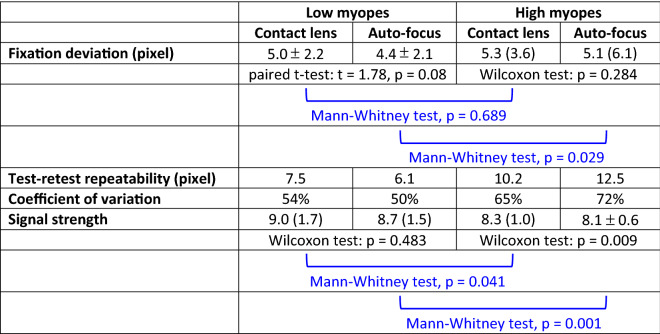
Fixation deviation and stability, signal strength and refractive astigmatism of low and high myopic groups. Results are presented as mean ± SD, or median (IQR).

**Figure 2:**
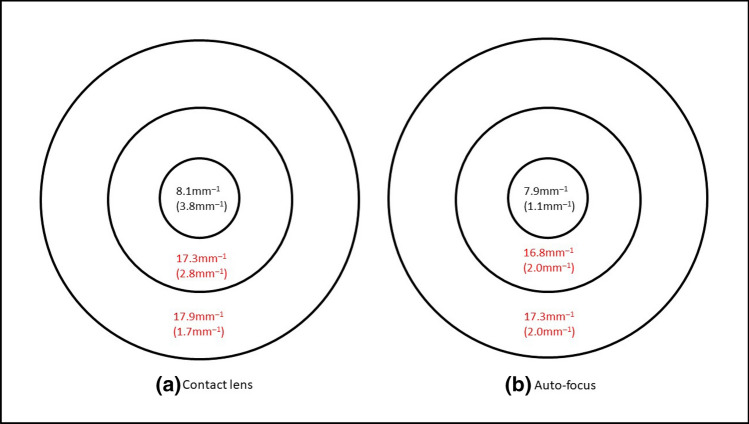
Vessel length density of 74 eyes when corrected with (**a**) contact lens and (**b**) the auto-focus system. Results are expressed as median (interquartile range) using the ETDRS central circle, inner and outer rings of the 6 mm grid. Significant difference between the two correction methods is marked in red.

**Figure 3:**
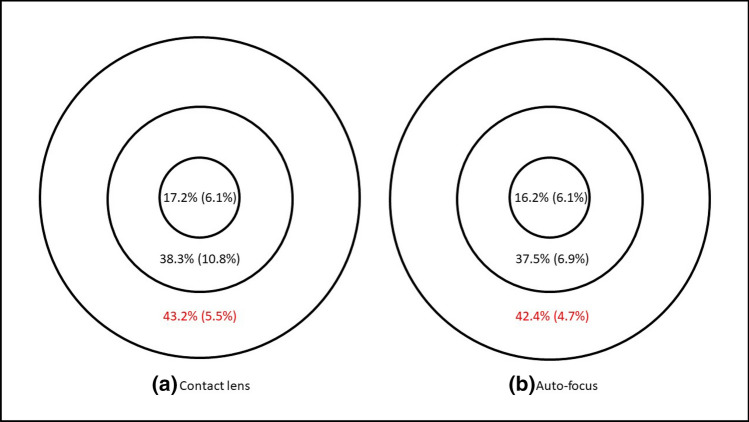
Perfusion density of 74 eyes when corrected with (**a**) contact lens and (**b**) the auto-focus system. Results are expressed as median (interquartile range) using the ETDRS central circle, inner and outer rings of the 6 mm grid. Significant difference between the two correction methods is marked in red.

Considering all 74 eyes, VD was higher when corrected with contact lenses in the ETDRS outer ring (median: 17.9 mm^−1^ vs 17.3 mm^−1^; Wilcoxon: p = 0.005), and inner ring (median: 17.3 mm^−1^ vs 16.8 mm^−1^; Wilcoxon: p = 0.028), Fig. 2. PD was also higher when corrected with contact lenses in the ETDRS outer ring (median: 43.2% vs 42.4%; Wilcoxon: p = 0.003), Fig. 3. FAZ area was similar between contact lens correction (0.251 ± 0.097mm^2^) and auto-focus (0.250 ± 0.102mm^2^), paired t-test, t = 0.108, p = 0.91.

**Figure 4:**
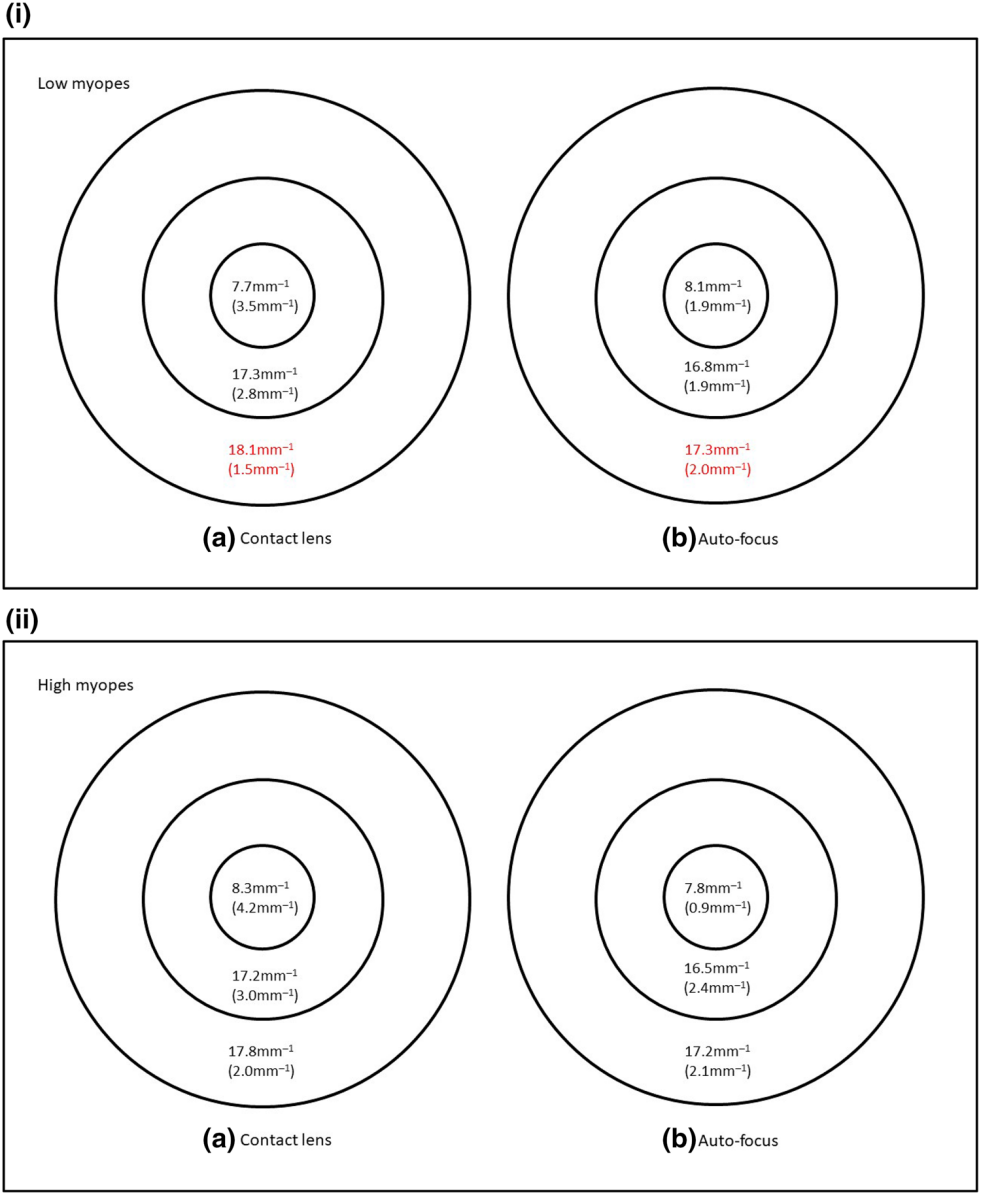
(**i**) Vessel length density of 34 eyes from low myopes when corrected with (**a**) contact lens and (**b**) the auto-focus system. Results are expressed as median (interquartile range) using the ETDRS central circle, inner and outer rings of the 6 mm grid. Significant difference between the two correction methods is marked in red. (**ii**) Vessel length density of 34 eyes from high myopes when corrected with (**a**) contact lens and b) the auto-focus system. Results are expressed as median (interquartile range) using the ETDRS central circle, inner and outer rings of the 6 mm grid.

**Figure 5:**
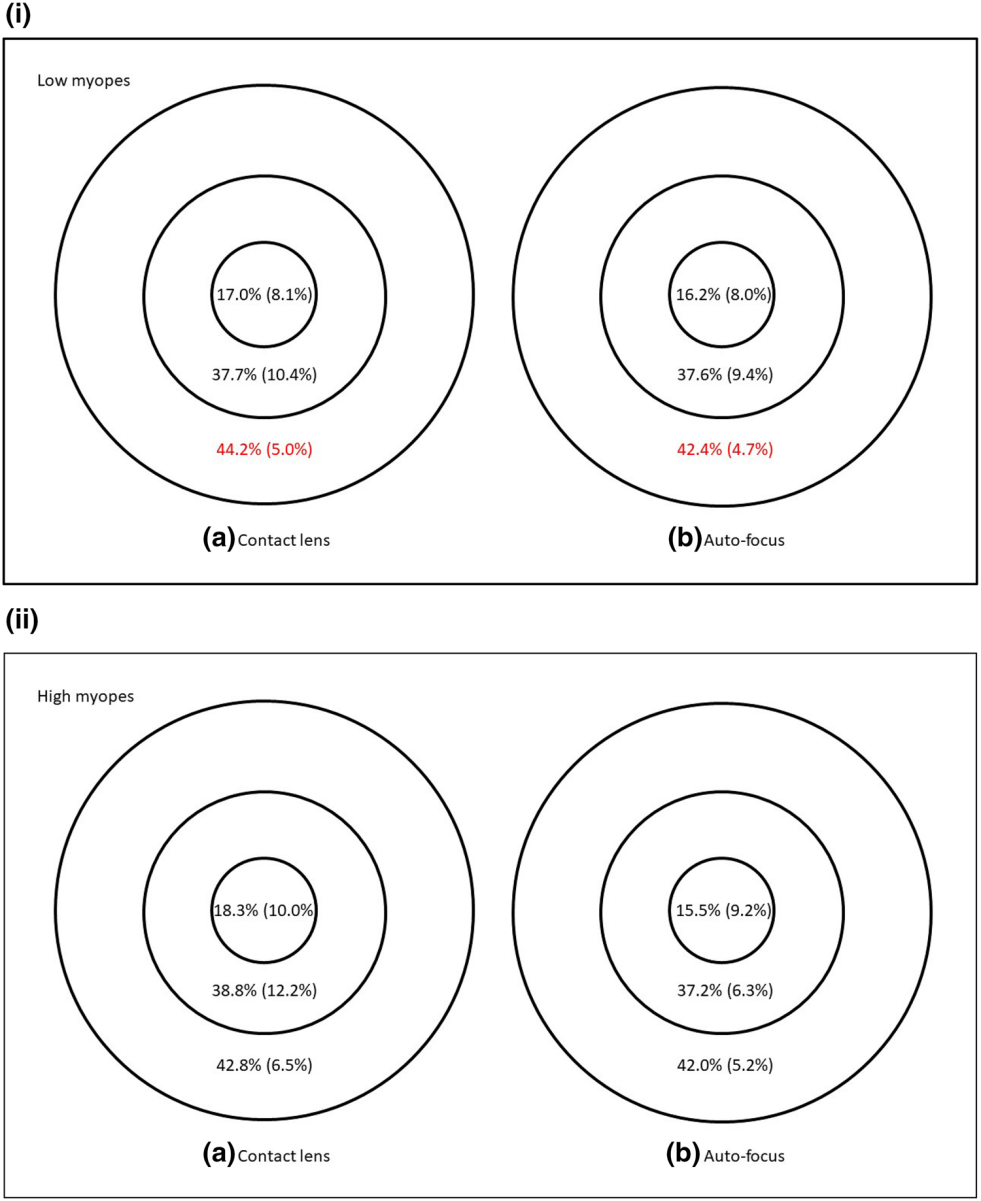
(**i**) Perfusion density of 34 eyes from low myopes when corrected with (**a**) contact lens and (**b**) the auto-focus system. Results are expressed as median (interquartile range) using the ETDRS central circle, inner and outer rings of the 6 mm grid. Significant difference between the two correction methods is marked in red. (**ii**) Perfusion density of 34 eyes from high myopes when corrected with (**a**) contact lens and b) the auto-focus system. Results are expressed as median (interquartile range) using the ETDRS central circle, inner and outer rings of the 6 mm grid.

Comparing individual refractive groups, contact lens correction yielded a slightly higher VD in the ETDRS outer ring in low myopes (median: 18.1 mm^−1^ vs 17.3 mm^−1^; Wilcoxon: p = 0.031) but no significant difference in other regions. VD was similar using the two correction methods in high myopes, Fig. 4. Contact lens correction gave a slightly higher PD in the ETDRS outer ring in low myopes (median: 44.2% vs 42.4%; Wilcoxon: p = 0.006). PD was similar in high myopes from the two correction methods, Fig. 5. FAZ area was similar between contact lens correction (0.251 ± 0.091mm^2^) and auto-focus (0.247 ± 0.100mm^2^) in low myopes (paired t-test, t = 0.326, p = 0.75), and in high myopes (contact lens correction: 0.251 ± 0.105mm^2^; auto-focus: 0.253 ± 0.106mm^2^, paired t-test, t = − 0.135, p = 0.89).

## Discussion

This study has revealed that high myopes had better fixation stability during OCTA measurement when soft contact lenses were worn (TRR: 10.2 pixels, CV: 65%), compared with using the built-in auto-focus system (TRR: 12.5 pixels, CV: 72%). However, low myopes had better fixation stability than high myopes regardless of correction method used (Table 2). Regarding fixation deviation, it was similar regardless of correction method in each group. Clinically, it is common to acquire OCTA images using the built-in auto-focus system to compensate for patients’ refractive errors. Fixation deviation was found to be significantly greater in high myopes when compared with low myopes using the built-in auto-focus system. Post-hoc power analysis, using G*Power (version 3.1.9.4, Dusseldorf, Germany), determined that the sample size had 86% power (α = 0.05, one-tailed) to detect an effect that exists.

**Figure 6:**
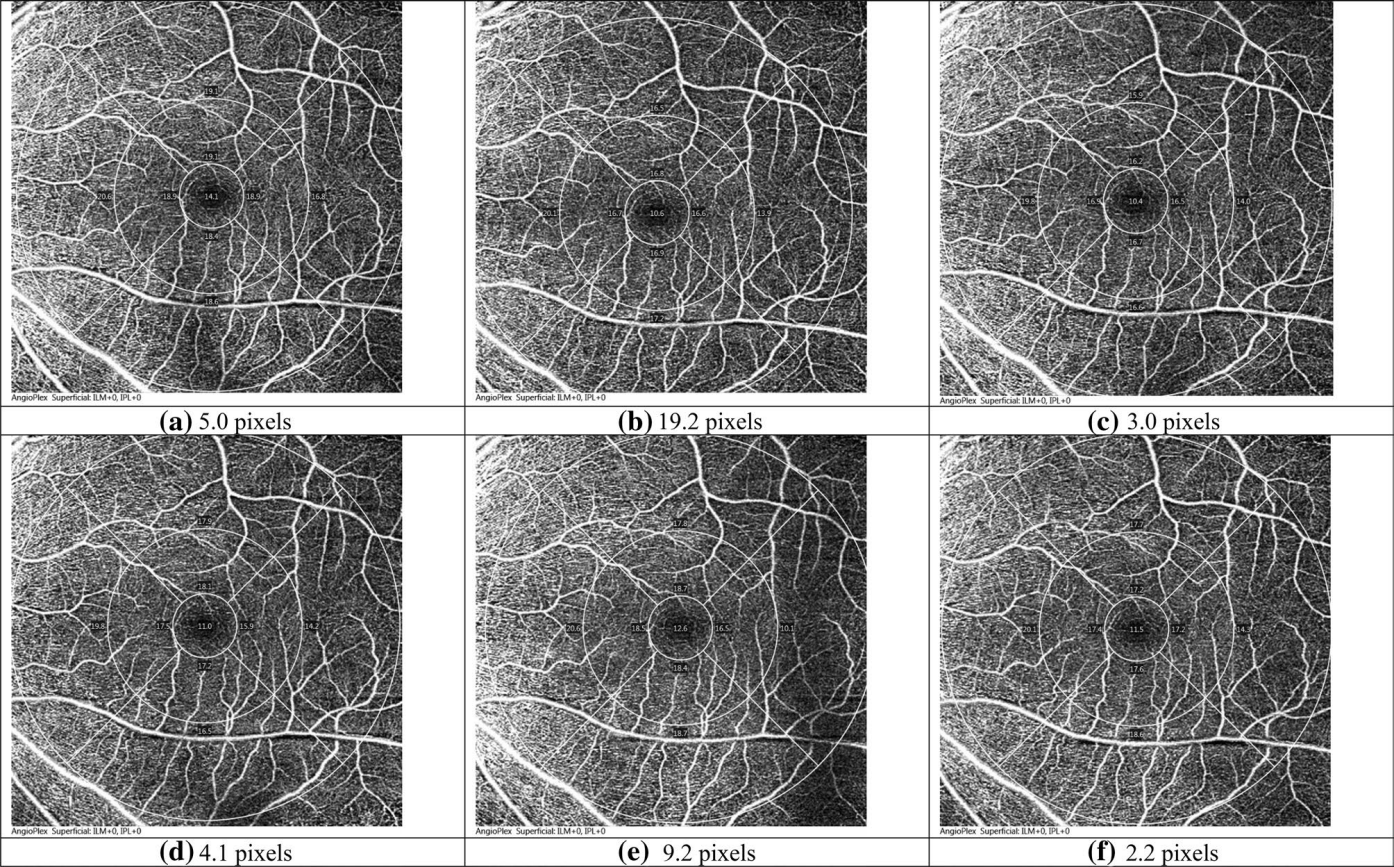
Three OCTA images from subject #027. (**a**–**c**) Correction with auto-focus (mean FD: 9.1 pixels; TRR: 8.8 pixels); (**d**–**f**) Correction with contact lens (mean FD: 5.2 pixels; TRR: 3.6 pixels).

**Figure 7:**
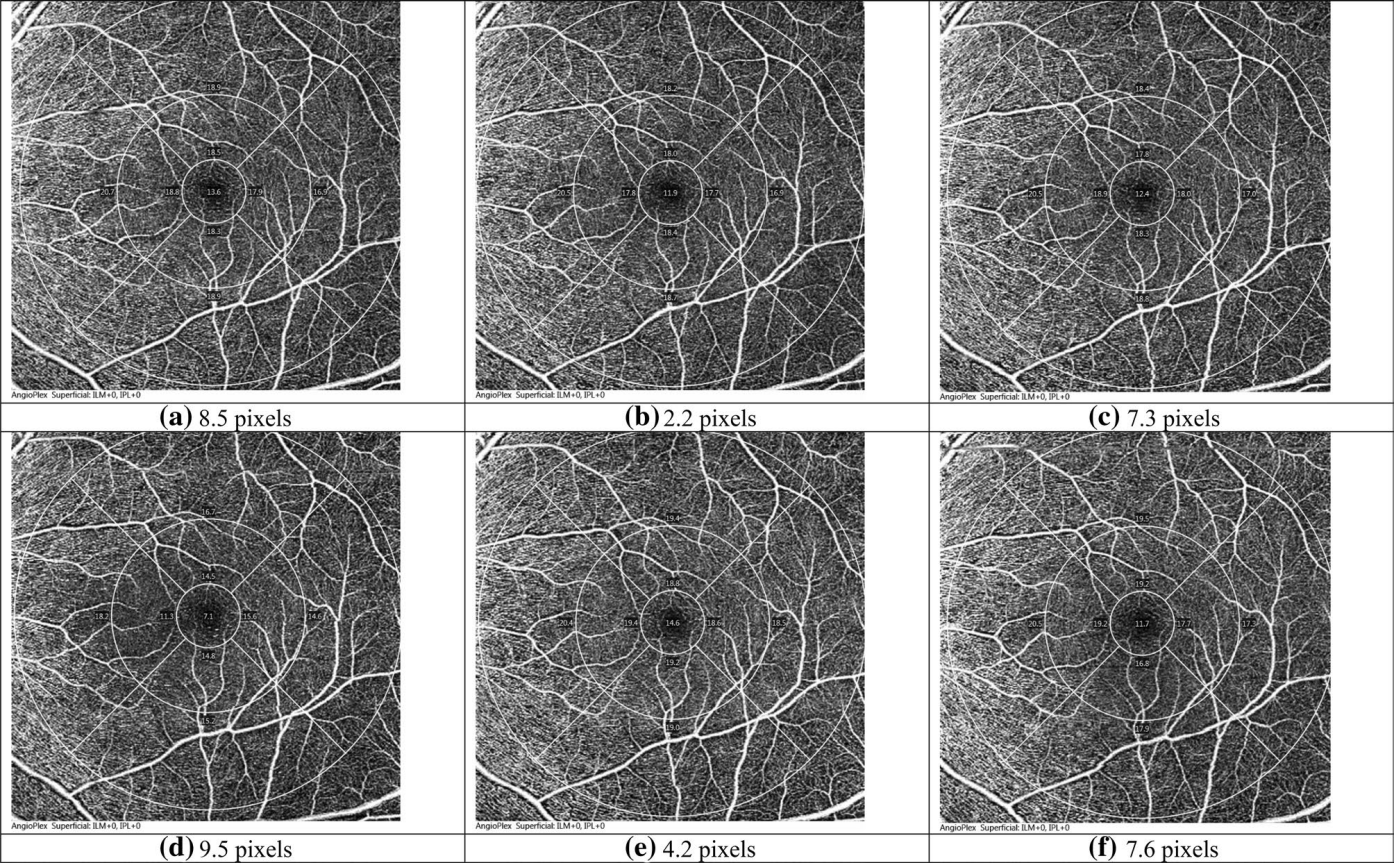
Three OCTA images from subject #032. (**a**–**c**) Correction with auto-focus (mean FD: 6.0 pixels; TRR: 3.3 pixels); (**d**–**f**) Correction with contact lens (mean FD: 7.1 pixels; TRR: 2.7 pixels).

The implications of fixation deviation and test–retest repeatability are different. A poor test–retest repeatability means that the scanned areas varied a lot in sequential measurements. A great fixation deviation means that the fovea is not at the center of the grid. For the latter, one or two ETDRS sectors could have smaller scanned areas compared with the others. However, it could still have good test–retest repeatability from consecutive measurements. Test–retest repeatability could be more important. Two examples can be used to illustrate this. Figure 6 is OCTA maps of the left eye from three consecutive measurements of a high myope, of which Fig. 6a–c were results using the auto-focus mode. Fixation deviation from each measurement varied differently (from 3.0 pixels to 19.2 pixels, mean of 9.1 pixels). The test–retest repeatability was 8.8 pixels. Figure 6d–f were results using habitual contact lens correction, in which fixation deviation in each measurement was less (from 2.2 pixels to 9.2 pixels, mean of 5.2 pixels) and the test–retest repeatability was smaller, 3.6 pixels. Both the mean fixation deviation and TRR were better using contact lens correction. Figure 7 shows OCTA maps of the left eye from three consecutive measurements of another high myope. Although mean fixation deviations were similar in the two correction modes (6.0 and 7.1 pixels), fixation deviations from three consecutive measurements were all towards the same superior temporal region, which resulted in a small TRR (3.3 and 2.7 pixels).

From our knowledge, this is the first study to investigate the effect of fixation deviation and stability during OCTA measurements. Clinically most practitioners acquire just one OCTA measurement because there is no averaging function from any proprietary software. It is important to have the same measurement area in follow up visits for accurate comparison and diagnosis. Taking Fig. 6 as an example, if an examiner relies on Fig. 6a, the outer ring at the temporal sector was cropped. The cropped areas were outer rings of the nasal and inferior sectors in Fig. 6b. To tackle this problem, a practitioner can put the ETDRS grid to the center of the 6 × 6 mm scan area. This is not normally done because the fovea may not be at the center of the 6 × 6 mm scan area.

A 6 × 6 mm scan has 350 × 350 pixels. The transverse resolution is 17.2 μm. A 3 × 3 mm scan has a higher resolution of 12.2 μm, but the scan area is smaller. The test–retest repeatability in high myopes was 12.5 pixels when using the auto-focus system, which was improved to 10.2 pixels when corrected with soft contact lenses. This is equivalent to a variation of 215 μm and 175 μm, respectively. This order of variation would result in significant measurement errors in OCT^14,15^.

Signal strength was found better in low myopes (Table 2). Previous studies have demonstrated impact of low signal strength on OCTA measurements^20^. The signal strength was set at 7 as the criterion and the averaged signal strength was above 8 in all four conditions. Lim et al.^16^ compared effect of signal strength on OCTA metrics using the same Cirrus OCTA device. VD and PD increased with a higher signal strength. There was no significant difference in VD and PD between signal strength of 9 and 10. Lee et al.^21^ also used a Cirrus OCTA device and found that repeatability (in terms of coefficient of variation) improved even when signal strength was slightly higher from 9 to 10. OCTA metrics were found increased when signal strength was 9 compared with signal strength of 8. Each OCTA device has its own proprietary algorithms. Yu et al.^10^ found that vessel density from AngioPlex was more influenced by signal strength compared with AngioVue.

It is important to have good fixation stability to ensure similar ETDRS subfields are measured in consecutive OCTA measurements. To achieve this, myopes with good CL-VA can continue wearing their contact lenses during OCTA measurements. This is more important in subjects with refractive astigmatism corrected with toric contact lenses (Table 2). The lack of astigmatic correction in the built-in auto-focus system might result in greater variation of fixation deviation (defined as fixation stability in the current study) in patients with astigmatism. Berkenstock et al.^11^ corrected their patients with soft contact lenses based on spherical equivalent even for astigmatic refractive errors. Optimal refractive correction is important in various ophthalmic procedures, such as kinetic perimetry^22^, OCT^8,23^, and OCTA^9^. Youm et al.^24^ compared retinal nerve fiber layer (RNFL) thickness measurement with and without soft contact lens wear. Although they found that RNFL was thicker without soft contact lenses, they used old version time-domain OCT with poor resolution.

In general, little difference in OCTA metrics was observed via two different correction methods (Figs. 2, 3, 4, 5). This could be due to the protocol using an average from three OCTA measurements. Previous studies found that averaging OCTA images could improve vessel clarity that might enhance accurate acquisition of OCTA metrics^13,25^. Uji et al.^26^ reported that averaging three OCTA frames resulted in significant improvement, while averaging five frames could result in almost identical findings. Clinically, automated averaging of several OCTA metrics for an overall result is not available from any proprietary software. In addition, it may not be feasible to acquire too many OCTA images. Our recent study found that OCTA metrics from averaging three OCTA measurements could reach similar VD and PD compared with averaging five measurements^19^. Considering that OCTA is more commonly performed in patients with a compromised retina and, hence, with poor vision, taking several OCTA measurements may be advisable.

There are different OCTA devices which are not interchangeable^27^. Therefore, patients must be reviewed using the same machine for accurate longitudinal monitoring. Our institute has both the Cirrus (Cirrus 5000 HD-OCT; Carl Zeiss Meditec, Inc, Dublin, California, USA) and Spectralis (Spectralis; Heidelberg Engineering, Heidelberg, Germany) systems, which use a split-spectrum amplitude-decorrelation algorithm and a probabilistic OCTA algorithm, respectively. The Cirrus system was found to be superior to the Spectralis system in terms of fewer artefacts and better repeatability^28,29^. The operation manual from Spectralis advised examiners to use glasses or contact lenses for patients with high astigmatism in order to enhance image quality. From the current findings, contact lens correction for OCTA measurements is recommended, especially for high myopes.

## Limitations

There are some limitations in this study. Firstly, types and powers of soft contact lenses worn by the subjects were not controlled. Previous studies underwent RNFL thickness^30^ or optic nerve head (ONH) measurements^31^, using time-domain OCT through the same soft contact lens material, but different lens powers. These investigations did not find significant differences in optic nerve head parameters. Lee et al.^32^ used soft contact lens of different powers by spectral domain OCT and found similar central macular thickness results. Sampson et al.^33^ explained in detail how important ocular magnification affects OCTA metrics. Unfortunately, this confounding factor was rarely considered in OCTA studies^34^. It is important to consider the magnification effect^35^, especially in highly myopic eyes^36^. In the current study, pixel rather than linear distance was used to evaluate fixation deviation and stability. Another limitation of using Cirrus OCTA was the lack of automated deep capillary plexus OCTA metrics. This could be due to the challenges and difficulties in quantifying deep capillary plexus in terms of projection artefacts^37^. However, third-party software such as ImageJ (National Institutes of Health, available at https://imagej.nih.gov/ij/download.html) could be as good as built-in proprietary software^29^. Rao et al.^17^ compared difference in peripapillary PD and flux index with and without referencing to a baseline scan using the “track to prior scan” option. Coefficient of repeatability improved when referring to a baseline scan in subsequent scans. We did not use this option to make each OCTA scan an independent one. Practitioners are advised to use this option if they plan to average multiple OCTA scans to generate an average result. Finally, the current study only included healthy subjects with high myopia. Patients with poor vision due to ocular diseases could be more affected by fixation instability in OCTA.

## Conclusions

The study revealed that fixation deviation was similar between the two myopic groups when habitual soft contact lenses were worn. It could be due to the similar contact lens corrected VA of the two groups. High myopes had better fixation deviation and stability through contact lens correction. This confirmed the study’s hypothesis that high myopes had more stable fixation when corrected with contact lenses.
